# Modifications to consent documentation with adults with communication disorders following brain injury: An exploratory study

**DOI:** 10.1111/dewb.12458

**Published:** 2024-07-14

**Authors:** Jennifer Watermeyer, Chiara Aylward

**Keywords:** adult, brain injury, communication disorders, consent documentation, informed consent

## Abstract

Consent documentation for research studies is often inaccessible to people with neurogenic communication disorders following brain injury and there is limited literature on specific modifications for informed consent. This exploratory study aimed to identify effective strategies and modifications to consent processes for adults with brain injury. Using a fictitious research study, we developed a set of Participant Information Sheets (PISs) varying in complexity, presentation format, and communication modality. Evaluations were conducted with eight participants. Findings indicated diverse participant preferences for PIS modifications, suggesting simplified vocabulary, reduced text, carefully selected images, and an interactive presentation modality as helpful strategies. Building on previous literature, we present refined guidelines for consent modifications for adults with neurogenic communication disorder after brain injury. These guidelines can promote more appropriate inclusion of communicatively impaired populations in research and assist ethics committees and researchers in preparing modified consent documents.

## 1 INTRODUCTION

The informed consent process in any research study is a crucial step towards upholding the autonomy of the individual. The process of consent should provide an individual with information that allows them to have a complete understanding of the risks and benefits of participating in a study and make a decision about participating. However, this process is often overlooked or bypassed when research involves vulnerable adults with neurogenic communication disorders (NCDs) following acquired brain injury (ABI). This study aims to promote the inclusion of adults with NCDs in research studies by offering a set of guidelines based on an exploratory study for making modifications to consent documentation that may be most helpful and acceptable to this population.

An NCD involves impairment in communication following injury, abnormality or disease in the brain or other part of the nervous system, typically after an ABI such as a cerebrovascular accident (CVA) or traumatic brain injury (TBI). NCDs may include aphasia (impaired comprehension and production of spoken and written language), dysarthria or apraxia (both being motor speech disorders that result in impaired speech production), right hemisphere disorders, and cognitive‐communicative impairments (disruption of cognitive skills such as memory, executive function and attention that impact communication).^1^

People with NCDs are commonly excluded from research studies due to the challenges involved in obtaining informed consent from these populations. In some instances, the assumption may be made that people with NCDs do not have the capacity to consent^2^ and a caregiver may be asked to give consent participation on their behalf.^3^ Alternatively, they may be included in studies where there is no modification to the consent process nor any support offered to promote understanding of information about the study, despite their cognitive‐communicative challenges.^4^

Information about a research study is typically provided in the form of an Informed Consent Form or a Participant Information Sheet (PIS), also referred to as a Participant Information Leaflet. This is traditionally a written document and is separate from the form which participants sign to indicate their provision of written consent. The PIS should typically explain the study in layman's terms. Depending on the nature of the study, this document may be lengthy and may contain complex scientific jargon.

In the case of people with NCDs, information about a study needs to be provided in a modified format (or formats) that promotes comprehension despite the presence of cognitive‐communicative challenges. There are some evidence‐based guidelines available for modifications to consent documents for people with NCDs, particularly for people with aphasia.^5^ However, some suggestions given in the literature refer to modifications to literature for leisure reading or health information documents^6^ and some studies focus on experiences of obtaining consent rather than on actual evaluations of modified consent documentation with vulnerable participant groups.^7^ There are a few articles that focus on consent modifications for TBI,^8^ but these primarily speak to different types of consent required rather than to modifying consent documentation. Importantly, many of these studies do not include English Additional Language (EAL) speakers and most do not involve evaluations of PIS formats that include alternatives to written options such as video or PowerPoint.

Suggestions for modifying consent documents for people with NCDs include using graphics or pictograms (especially computer‐generated images),^9^ reducing information to a single question per page, using bolding for emphasis, using a large font, and reducing the length of sentences.^10^ Importantly, Jayes and Palmer^11^ caution that some adults with NCDs may find modifications to PIS documents, especially pictures, insulting. Thus, it is important to determine which types of modifications may be more acceptable as well as helpful in terms of promoting understanding for people with NCDs.

It is important that such research also considers the perspectives of adults with NCDs who are English Additional Language (EAL) speakers, since many consent documents are presented in English. A language barrier may further restrict a potential participant's ability to understand these documents. Furthermore, cultural differences between researchers and participants require researchers to be mindful of making use of culturally appropriate and relevant resources and images within informed consent documents to prevent misunderstandings.^12^ There is limited research that involves people with NCDs who are EAL speakers, apart from Penn et al.'s^13^ study.

The main aim of our study was to determine the types of modifications to informed consent documentation that may be most and least acceptable and helpful to assist adults with NCDs to understand the information provided about a research study.

## 2 MATERIALS & METHODS

### 2.1 Study design

Our research question was: What strategies and modifications to consent processes are most helpful and acceptable to adults with NCDs following ABI for promoting understanding of information about a research study?

A qualitative descriptive design was adopted for this exploratory study. We created a set of modified PIS documents for a fictitious research study (a drug trial) using different modalities that may assist people with NCDs to understand information about a research study. Our modifications were informed by existing recommendations in the literature. We then presented each of these PIS formats to a sample of adult participants presenting with NCDs, observing how they responded to each one and informally interviewing them about their preferences and the (perceived) effectiveness of each PIS in aiding their understanding of the fictitious study. These exploratory sessions were video recorded.

Frankel's^14^ trial was selected as a fictitious example for our study due to the multi‐stage, randomized, double‐blind nature of the trial which included complex concepts such as *baseline, active, placebo* and *withdrawal phases*. Her research investigated the response of a group of stroke survivors to pharmacological therapy on a one‐month trial of Leviteracetam (Keppra®). Jennifer is an author of a previous paper^15^ which explored the informed consent process for this same trial. Frankel's study was conducted in the same city, and we recruited participants from the same ABI support group she did, ensuring that the context and participant profiles of her drug trial closely matched those of the current study.

### 2.2 Ethical considerations

Ethical clearance was obtained from the University's Human Research Ethics Committee (HREC) Non‐Medical (clearance number STA_2022_11). Informed consent was obtained from participants using a simplified PIS incorporating graphics and a simplified consent document (based on guidelines in Penn et al.^16^).

To ensure that participants did not become confused and believe they would really be participating in the fictitious research study, during the data collection process participants were given frequent verbal reminders that the information presented in the PIS documents was in fact fictitious. More than half of the participants became confused during their interviews and required reminders of the fictitious nature of the study being presented to them and/or a reexplanation of the purpose of the current research study. Participants were also regularly reminded of the purpose of the research, which was to comment on which modifications were helpful or acceptable. The video recordings of the process were shared between the two authors only.

### 2.3 Participants and recruitment strategy

A convenience sample was recruited via a non‐probability purposive sampling strategy from a support group for people with ABI in Johannesburg. The exploratory nature of the research study did not limit the study to a particular type of ABI (e.g. CVA or TBI).

Participants were required to self‐identify as fluent in English as the PIS documents were prepared in English. Individuals were excluded when their communicative impairments were so significant that they were unable to sufficiently comprehend the PIS documents and participate in the interviews. We worked closely with facilitators at the ABI support group who were familiar with potential participants and could advise on their suitability for participation in this study.

A total of eight participants with mild to moderate NCDs after ABI were recruited, in line with Clarke and Braun's^17^ recommendations for a small exploratory project. Demographic variables for the sample are included in Table 1.

**Table 1:** Demographic details of participants.

Participant number	Age (years)	Gender	Type of ABI†	Observed communication challenges	Time since onset (yrs)	Home language	Additional language	Level of education	Employment status	(Previous) employment	Reported communicative strengths	Reported communicative challenges
1	32	M	CVA‡	Mild cognitive impairment Dysarthria	8	Afrikaans	English	college diploma	unemployed	personal trainer	literacy, comprehension	dysarthic speech
2	52	M	CVA	Dysarthria	13	English	Afrikaans	college diploma	employed	debt collector	literacy, comprehension	dysarthic speech
3	54	M	CVA	Hemi‐spatial neglect Dysarthria Moderate cognitive impairment Memory impairment Word finding difficulties	3	English	‐	college diploma	unemployed	filmmaker	comprehension	dysarthic speech literacy
4	41	M	CVA	Severe apraxia of speech	11	English	Siswati	university degree	unemployed	business analyst	literacy, comprehension	apraxia of speech
5	36	F	TBI§	Moderate cognitive impairment Memory impairment	6	Sepedi	English	Grade 11	unemployed	printer	literacy, speech	comprehension memory
6	43	M	TBI	Moderate cognitive impairment Memory impairment Dysarthria Word finding difficulties	12	Setswana	English	Grade 12	unemployed	tavern owner	literacy, comprehension	dysarthic speech memory
7	34	M	TBI	Moderate cognitive impairment Memory impairment	13	isiZulu	English	Grade 10	unemployed	student	literacy, comprehension	memory
8	54	M	TBI	Mild cognitive impairment Dyslexia	13	English	Afrikaans	college diploma	unemployed	information technology manager	comprehension, expressive language	dyslexia

^†^ Acquired Brain Injury

^‡^ Cerebrovascular Accident

^§^ Traumatic Brain Injury

Most participants were male (n = 7) while only one was female (n = 1) – which aligns with contextual trends showing a higher incidence of TBI amongst males,^18^ but does not align with trends showing that females have a higher risk for CVA than males.^19^ The mean age of the participants included in the study was 43.25 years old. Three participants fell within the 30‐39 age group. Two participants were between the ages of 40 and 49. The remaining three participants were between the ages of 50 and 55 years old. Half (n = 4) of the participants had suffered a CVA, whilst the other half (n = 4) had sustained a TBI. The mean time since ABI was 9.9 years.

Six participants reported that literacy (reading and writing) was one of their communicative strengths. Two participants reported literacy as one of their weaknesses: one participant presented with right‐sided hemispatial neglect^20^ after CVA, while another had premorbid dyslexia (a learning disorder involving difficulty reading). Most participants (n = 7) reported language comprehension as a strength. Five participants cited clarity of speech as a communicative challenge. Of these, four participants presented with dysarthria and one presented with apraxia of speech. Two participants stated that memory was one of their challenges. We did not have access to formal test scores for each participant and we decided not to formally test participants' cognitive‐communicative skills, focusing instead on reported strengths and weaknesses and observed functional behaviours during the research tasks.

Half of the participants (n = 4) reported English as their home language and half (n = 4) described themselves as English Additional Language speakers. Most participants (n = 7) included in the study were unemployed and one was employed on a part‐time basis. Half of the participants (n = 4) had completed a college diploma, one had completed a university bachelor's degree and the rest had completed varying levels of high school education.

### 2.4 Research instruments

Although consent documentation for a drug trial is typically very detailed and lengthy, for the purposes of this study we decided to keep the information as brief as possible. We developed six different formats for presenting information about the study using different modalities, as indicated below. (These are included as Supplementary Materials to this paper.)

1. A paper‐based PIS prepared using the University's HREC guidelines for the preparation of a PIS (with a Flesch‐Kincaid grade level score of 10) of one page in length.
2. A paper‐based PIS with language simplified to a basic readability level with a Flesch‐Kincaid grade level score of 6.5. (Although this figure is below local guidelines of a maximum grade level of 8 for lay persons,^21^ we acknowledge that this score is still relatively high for adults with NCD). This document was one page in length.
3. A paper‐based PIS with language simplified to a basic readability level, also including italicization, headings, highlighting and bolding of key information, of two pages in length.
4. A paper‐based PIS simplified to a basic readability level using three options of graphic formats (line drawing, cartoonised drawing, photographic image) presented side by side to illustrate key points. This document was seven pages in length.
5. A PowerPoint presentation giving information about the study using simple language and graphics. This was presented on an iPad, allowing participants to move through slides independently. The presentation was 20 slides long.
6. A video recording of the researcher explaining the study, with subtitles and graphics. This was presented on an iPad and was just over four minutes long.

Our selection of the PIS formats was informed by relevant literature. In Pearl and Cruice's^22^ article, suggestions such as reducing sentence length and using fewer complex sentences led to the creation of PIS #2. Their suggestions for the use of bolding, italicizing, and bullet points informed the development of PIS #3. Penn et al.^23^ suggest using colour illustrations to assist individuals with aphasia with understanding information, which informed the creation of PIS #4 and #5. Pearl and Cruice^24^ also mention the use of video and PowerPoint, which informed the creation of PIS #5 and #6.

A set of basic questions was developed to guide the discussion of each PIS format presented and to facilitate a reflective general discussion of each participant's experiences of the set of materials presented. These questions focused on modifications that were helpful or not helpful in promoting understanding, the most acceptable or preferred formats, and suggestions for improving the consent process.

### 2.5 Data collection

Chiara facilitated the data collection, which took place face‐to‐face in a quiet room either at the support group premises or in participants' homes. Participants were asked a series of demographic questions to determine factors that may impact their understanding of the PIS documents and the suggestions they made to modify them. These questions related to their diagnosis, level of education, home language, and strengths and weaknesses in communication skills.

The set of PIS documents was then presented in an order based on each participant's reported strengths and weaknesses in cognitive‐communicative skills as well as the engagement with each participant during the consent process before data collection. For example, participants who reported weaknesses in reading were presented with PIS #6 initially. However, participants who reported reading and comprehension as strengths were given PIS #1 or #2 initially. Each participant was shown all the PIS documents; however, they were not required to read through the written PIS documents (PIS #1, #2 and #3) if they were unable to do so. When PIS #4 was presented, participants were asked to select their preferred graphic format with reference to the corresponding statement in the PIS. After all PIS formats had been presented, participants were asked to comment on which format(s) they found most acceptable. Participation in the study lasted between 45 minutes to one hour and 30 minutes (average = 50 minutes).

Each PIS was presented to the participant, followed by a discussion about that particular format. This entire process was video recorded and supplemented with notes on how participants responded to each format presented. Unstructured observations were used to observe participants' verbal and non‐verbal behaviours (including facial expressions, tone of voice, and use of gestures) while engaging with each PIS.

Participants' knowledge and understanding of the information presented via the PIS documentation were informally assessed using a teach‐back method^25^ and through observation of verbal and non‐verbal responses. We did not conduct a scored assessment of knowledge following the presentation of each PIS format, because there would likely have been a testing or rehearsal effect due to repetition of the information presented during data collection.

### 2.6 Data analysis

Chiara watched each recorded session and made notes on participants' responses to questions, comments, and opinions. Relevant quotes from the discussions were transcribed verbatim.

Information from the notes from both the observation of the videos and the observations conducted in real‐time during the research activities was triangulated. Cross‐case analysis^26^ was used to analyse the data obtained from the observations and interviews for each participant. Participant responses and researcher observations were tabulated for each case and compared to patterns exhibited in other cases to identify similarities and differences between the patterns. The information inserted into the tables was divided into three categories:

1. Accommodations and adaptations that worked in helping participants understand the fictitious research study;
2. Accommodations and adaptations that did not work in helping participants understand the fictitious research study;
3. Suggestions for how the informed consent process could be altered or improved to better accommodate participants' needs.

Common themes and trends that appeared across each session were noted, including differences and similarities between participants' responses. These were discussed between both authors and consensus on the findings was achieved.

### 2.7 Trustworthiness

Shenton's^27^ suggestions were used to promote trustworthiness in this study. Credibility was achieved via triangulation of data from the unstructured observations and semi‐structured interviews. Regular debriefings between the authors additionally ensured credibility. Transferability was encouraged through purposive sampling of data collected from a particular group of participants, a detailed description of methods used, and attempts to provide a thick description of the findings. Dependability was achieved through careful documentation of the research process in a research journal. Confirmability was achieved via an audit trail and careful checking and consolidation of the analysis by both authors.

## 3 RESULTS

In this section, we outline participants' responses to the different formats of PIS documents presented, including written information, graphics and multimedia options. We start with a description of general participant responses to the research tasks, including strategies used to support understanding of the information presented, and end with a description of overall trends and preferences noted across the data set.

### 3.1 General responses from participants

Following presentation of PIS #1, many participants claimed to have understood the information when asked but were unable to explain the study in their own words via a teach‐back strategy. This strategy allowed an opportunity to gauge the participants' true level of understanding of the information and address any gaps in their understanding.

Another way to gauge the level of understanding of the participants was through observation of non‐verbal communication cues such as facial expressions. For example, P3 displayed various non‐verbal reactions whilst watching PIS #6, such as raising an eyebrow and glancing at the researcher when the list of side effects was given. This demonstrated that he possessed a sufficient level of understanding of the information to be able to respond appropriately to it.

Some participants required repetition of concepts multiple times to fully comprehend them, especially more complex concepts. For example, P7 was unaware of what a capsule was and asked for an explanation of the meaning of the word and a picture to understand the concept. He and some other participants expressed a need for time and opportunities to ask questions and request clarification on topics they did not fully comprehend.

Participants with unintelligible speech or apraxia of speech required accommodations to communicate their answers to the interview questions and ask questions for clarification. It was helpful to provide these participants with a pen and paper to allow them to communicate through writing and they also made frequent use of hand gestures to communicate. To better accommodate these participants, yes or no questions were used so that participants could easily respond by nodding or shaking their heads. It was also beneficial to offer these participants choices when asking them open‐ended questions, as this allowed them to select one of the options without having to communicate verbally.

### 3.2 Responses to the presentation of written information

Many of the participants, including those who listed literacy skills as one of their strengths, expressed that they preferred to have the written PIS formats read to them or to read the written PIS together with the researcher.

Participants reported that the use of shorter paragraphs made the information presented in the PIS documents more comprehensible. Participants commonly reported that the use of highlighting was effective in drawing their attention to the important information within the document. The use of bullet points when listing information such as side effects of a medication or tasks that they will need to do as part of a research study was commonly described as helping to make the information more “straightforward.” P8 stated that PIS #3 “makes more sense” as it informs participants about “exactly what we are going to do and how we are going to do it” in a list format.

Most participants communicated that they felt PIS #1 and PIS #2 required excessive reading. P1 claimed that he only “skimmed through it” due to the large amount of information being presented to him (despite this being a one‐page document). Another participant stated that they would “lose interest in (the document) very quickly” because of the amount of reading required. P3 could not engage with these PIS documents to any extent due to his right hemispatial neglect which interfered with reading. When participants were presented with PIS #1 and #2 first, they were generally unable to paraphrase the information correctly or else omitted important information.

PIS #3 was disliked by some of the participants of the study. P7 indicated that it resembled “study notes” which were used in an educational environment. The combination of headings, highlighting, bolding, underlining and italicizing all used in a single document appeared to make this PIS format overwhelming for participants. Participants further commented that the document was “lengthy” and thus difficult to engage with.

In general, participants remarked that they appreciated that the participant information sheets stated both the advantages and potential disadvantages of participating in the fictitious research study and felt this would aid them in making more informed decisions about participating.

### 3.3 Responses to the use of graphics

Participants generally responded positively to the inclusion of graphics within the PIS documents. Participants preferred the use of photographs rather than cartoonised pictures or line drawings within the participant information sheets. More than half of the participants expressed that photographs were their favourite graphic format. Participants stated that photographs were the least abstract of the different graphic types included, and thus easiest for them to comprehend. The photographs were however scrutinized by several participants who claimed that the quality of the printing made the photographs difficult to see or that there was not enough contrast between the subject and the background in the photograph.

Some participants preferred the use of cartoonised pictures. They reported that this format was more exaggerated than photographs in terms of the facial expressions and body language depicted in the pictures, thus making these easier to understand. Whilst cartoonised pictures were the second most popular form of pictural representation, many participants found these to be “childish” in nature.

Line drawings were the least popular form of graphic representation amongst the participants, with no one selecting it as their preferred graphic format. P2 described the line drawings as being “lifeless.” P3 stated that the abstract nature of the line drawings could be “scary” if interpreted wrong. He indicated that the line drawing of the patient receiving a medical evaluation appeared to depict him suffering a heart attack. This incorrect interpretation could lead participants to believe that a heart attack is a possible side effect of the medication in the fictitious research study.

Participants commented on the relevance of some of the graphics to the correlating information. For example, P8 stated that if the text speaks about filling in “four forms,” the accompanying graphic should contain four forms, rather than just one. Similarly, P3 stated that the accompanying graphic should present two capsules only rather than one capsule or multiple capsules, as the information given discusses two capsules. P6 remarked that the photograph of the medications made them appear to be illicit drugs rather than medication, due to the colour and appearance of the medications depicted. Many participants preferred a graphic that correlated with each piece of information given to them.

Participants frequently mentioned that any graphics used should be as relevant and representational as possible to the information they are attempting to portray. P3 stated that for the graphics to be beneficial, “[they] need to be accurate.” Most participants felt that the graphics should include photographs taken by the researcher and should also depict the researcher. These would make the graphics more relevant to the information and give a clearer idea of the procedures involved in participating in the research. Commenting further on the contextual nuances of the graphics, participants generally preferred the use of cartoonised pictures when medications were depicted, but preferred the use of photographs when people were depicted.

### 3.4 Responses to multimedia presentation formats

Overall, participants found the PowerPoint presentation format in PIS #5 the most effective for assisting them to understand the study and the most appealing of the formats presented. The use of colourful images within the PowerPoint presentation was generally praised by participants. The presentation of information via an iPad was successful, as participants noted that they appreciated the structure of the presentation and having the ability to change the presentation slide themselves once they had taken the time to understand the information and look at the graphics.

Only two of the participants preferred PIS #6 – the video recording – and most participants rated it as one of their least favourite options. Many participants stated that the use of video, text, and graphics simultaneously was an excessive number of stimuli to process, making it difficult to concentrate on the information being communicated. Participants mentioned that the video moved too quickly for them to efficiently read the information, look at the graphics and absorb the information being presented. Participants with deficits in reading due to visual neglect or dyslexia were unable to view the full screen at one time or read through the information quickly. The video was too long which caused them to lose interest in the content after some time. P1 and P2 commented on the tone of the speaker in the video, with P1 stating “it was presented to me as if [it was assumed] I am not going to understand. As if I am a little child or something.” Participants preferred a more conversational tone to be used when explaining a research study rather than a slower speech rate with simplified language and shorter sentences. P3 and P4 remarked that the speaker not making eye contact with the camera whilst delivering the information was “distracting” and further made the video feel less conversational.

Many participants suggested that a live conversation between the researcher and the participant would be more effective than the use of a video. Other participants suggested that they would prefer to receive written information regarding the research in combination with an informative video. P4 suggested that the use of animated cartoons, rather than a still image of a cartoonised picture within the video, may be more helpful. He felt that the use of animations within the video would be more beneficial in aiding understanding compared to still images appearing on the screen during the video.

### 3.5 Most and least favoured formats in relation to impairment profiles

**Graph 1:**
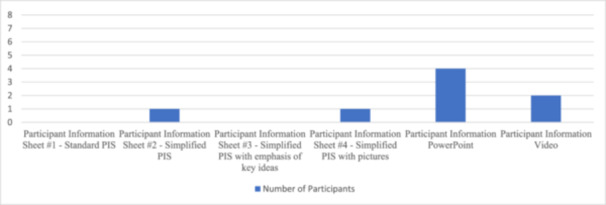
Participants' most favoured format for information about the fictitious research study.

**Graph 2:**
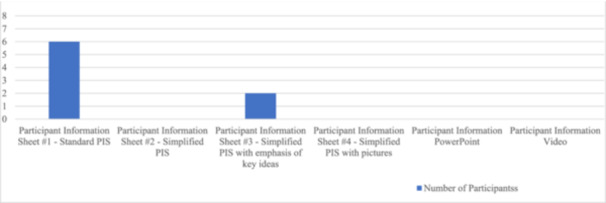
Participants' least favoured participant information formats.

Graphs 1 and 2 illustrate the most and least favoured PIS formats amongst the participant groups. The PowerPoint format of PIS #5 was judged as most helpful while the unmodified PIS #1 was judged as least helpful for promoting understanding of information about the fictitious research study.

Unsurprisingly, we noted that the participants' impairment profiles linked well with their preferences for PIS formats. For example, PIS #2 was preferred by P2, who described his literacy skills and comprehension abilities as strengths. P2 frequently interacts with written material as he is currently employed part‐time in a job that involves reading. His strengths evidently impacted his choice of PIS format. PIS #4 was preferred by P5, who described experiencing memory deficits. This PIS format may have been more helpful because it involved different modalities of information delivery. P5 experienced difficulty recalling information presented in the PowerPoint and video formats, but PIS #4 allowed P5 to reference back to the written and pictorial information at any point. Both participants with and without deficits in literacy skills preferred the PowerPoint format in PIS #5, making this format the most popular and arguably the most useful across a diverse set of impairment profiles in the participant group.

## 4 DISCUSSION

Modifications to the informed consent process are necessary when conducting research that involves adults with NCDs, to ensure that potential participants are supported in understanding information about a research study and consent is truly informed. Whilst recommendations have been made by various researchers for modifying written health information, educational documents, and other written documents, few researchers have studied in detail the kinds of modifications to consent documents that might be useful for people with NCDs after ABI.

Our findings suggest that modifying informed consent documentation for populations with NCDs is not as straightforward as simply adding graphics or changing the font size on a standard PIS. Instead, the modification process is a complex and highly nuanced one that should take the preferences of individuals with NCDs into account. Each modification made needs to be highly intentional and carefully planned – as Shiggins et al.^28^ suggest, “creating [NCD] inclusive research practices requires deliberate decisions that start at project conception and development”.

Previous studies researching modifications to a range of written documents for individuals with ABIs and NCDs have noted similar findings to ours. For example, Rose et al.'s^29^ study into design preferences for printed educational materials also found that photographs were the preferred form of graphic used in documents for individuals with NCDs. Pearl and Cruice's^30^ article provides detailed guidelines on the use of graphics, including that images used should be representative of the participants' age, race, and gender where possible. Our findings add more detail regarding the types of graphics that may be most useful for people with NCDs and their contextual preferences in this regard – for example, including photographs for people and cartoonised pictures for medications in consent documents. Rose et al.^31^ suggest the use of captions to accompany all graphics, due to the variety of meanings that can be inferred from a picture or photograph. Although we did not include captions with our graphics, our findings confirm that participants may not always interpret graphics in as straightforward a way as researchers may assume. Furthermore, Rose et al.'s^32^ finding that including a correlating graphic for each sentence or important point in a text can be beneficial may explain the success of our use of a PowerPoint presentation format, because this format provided a graphic for each point conveyed.

However, we noted some differences between our findings and some of the recommendations in the literature. For example, Shiggins et al.^33^ mention that a video with narration and pictures to explain a research study may be useful to some participants, but our findings show that this was not generally a preferred option and, in some cases, participants found the use of multiple modalities for information giving too overwhelming. We found only a few studies^34^ that mentioned the use of Powerpoint ‐ the most popular PIS format in our study ‐ but this tool was used differently in those studies compared to our study where participants could control the slide transitions on an iPad.

### 4.1 Implications

Despite the heterogeneity of our participant group in terms of cognitive‐communicative symptoms and preferences for modifications to consent documentation, we were able to identify several common themes and similarities across the general preferences of the participants, enabling the generation of a set of guidelines which we present in Table 2. These are combined with guidelines from previous literature exploring the modification of documents for individuals with acquired NCDs.^35^

**Table 2:** Guidelines for modifying Participant Information Sheets for adults with acquired NCDs.

**Layout**
**Amount of text** Reduce the amount of text presented on a page as far as possible. Keep documents as short as possible. **Paragraphs** Use short paragraphs focusing on one idea only. Make use of increased white space between each paragraph. **Font** Use a sharp and large font size (preferably San Serif font size 18+).
**Content**
Use shorter sentences with a maximum of 15 words each. Use simplified vocabulary and syntax.
**Emphasis of information**
**Highlighting and Bolding** Use highlighting to emphasize important phrases and bolding of text to emphasize important words. Do not use italics to emphasize information as this is more difficult to read. Do not combine multiple formats such as bolding and highlighting as this can make the document difficult to read. **Headings** Split up different chunks of information using appropriate headings. **Bullet Points** Use bullet points when listing information.
**Graphics**
In general, make use of photographic images rather than cartoonised pictures or line drawings. Be mindful of using graphics of medical procedures which can be frightening or easily misinterpreted. Consider using photographs for people and cartoonised pictures for medications. Ensure photographs are of the best possible quality and printed in high resolution. Ensure the subject of the graphic contrasts with the background to stand out sufficiently. Use colourful photographs to capture attention. Ensure photographs are as relevant to and as representational of the information they correlate with as far as possible. Ensure that people depicted in graphics are representative of the potential participant(s) and the researcher(s) in terms of age, race, and gender. Graphics depicting the researcher should be photographs of the researcher. Where possible, use photographs taken by the researcher(s) to depict concepts in a contextually grounded, accurate manner. Caption each graphic included to prevent misinterpretation of the image.
**Modality**
Where possible, offer information about a research study in a range of formats. Use multiple modalities to present information, e.g. present both a written document and a digital presentation in case of impaired understanding via one modality. If using a digital option, ensure this is interactive, i.e. the potential participant can turn a page or change a slide themselves on a computer or iPad. Consider accessibility options for digital modalities for participants presenting with a physical disability.
**Researcher Strategies**
**Check Understanding** Use a teach‐back method by regularly asking potential participants to paraphrase the information provided to determine understanding. This approach will reveal whether the potential participant needs more repetition or an alternative method of presentation. Observe potential participants' non‐verbal communication cues and responses to information presented, such as facial expressions, tone of voice and gesture, to gauge understanding. **Periodic Review** Allow opportunities for potential participants to ask questions and request clarification throughout the consent process. Repeat important concepts multiple times during the consent process. **Accommodations** Encourage the use of a range of facilitatory communication strategies including non‐oral communication from potential participants such as via gestures and writing. Read PIS documents aloud to the potential participant whilst pointing to the text being read so they can follow along. **Presentation of Information** Use a conversational tone when presenting information verbally. Do not make assumptions about how much or how little a potential participant understands. Be careful not to use a condescending or patronizing tone when presenting information to a potential participant.

Whilst our suggestions provide some general guidelines for modifying the informed consent process, it is important to keep in mind that such modifications should not be applied using a “one size fits all” approach. Participants with ABI have differing strengths, weaknesses, and experiences which affect their preferences for and responses to modifications to documents.^36^ Any modifications should be supported by research and take into consideration the needs of the individual participant.

“Where possible, people with [NCDs] should be offered a choice of information formats and also should be involved in the design of research information materials”.^37^ Speech‐language therapists working with adults with NCDs have a role to play here alongside researchers in ensuring that informed consent documents are suitably modified and thus accessible to potential participants.

Importantly, our findings have application to research with other populations and with the general public. In any study, researchers have a responsibility to ensure that information is presented to potential participants in an accessible, simple manner. The guidelines we present in this article may assist researchers in making consent documentation more accessible to participants in a range of contexts, particularly in instances where linguistic and cultural differences are present.

## 5 CONCLUSION

This exploratory study offers a suggested set of detailed and refined guidelines for consent modifications for individuals with NCDs, based on our findings and relevant literature. These guidelines may promote and facilitate better comprehension of complex information about research studies by people with NCDs and allow researchers to incorporate such individuals into their research in more ethical ways. The guidelines can also assist ethics committees in advising researchers about how to modify consent documents for people with NCDs.

### 5.1 Limitations

This exploratory study included a small sample size and the participants from the study were obtained from only one support organization which arguably limits the representativeness and generalisability of the findings. Although there were evenly matched numbers of participants with CVA and TBI included in our study, the participant group was heterogeneous in terms of presentations of sequelae of brain injury and cognitive‐communicative strengths and weaknesses. This made their requirements for modifications to documents and opinions on modifications equally as diverse. We did not directly assess participants' cognitive‐communicative skills in this study, relying instead on self‐reported and observed strengths and weaknesses in these areas. Again, this may limit the generalisability of the findings. The sample was skewed towards males, with seven of the eight participants being male. Although this aligns somewhat with ABI statistics, the perspectives of females presenting with NCDs were not widely included in this study and we acknowledge that females may present with differing opinions on the PIS modifications we presented. We acknowledge that conducting evaluations of PIS formats in one session may have led to fatigue amongst some participants and thus impacted the findings of the study, although it should be noted that this was not evident during data collection. Fatigue may have led participants towards a preference for the consent forms presented earlier in the session. In addition, our attempts to match participants' cognitive‐communicative symptoms to an appropriate PIS format may also have skewed the findings.

### 5.2 Recommendations for future research

Further research is needed on this topic to confirm and build on the findings of this exploratory study. Given that ABI may affect language domains to different degrees, it would be useful to explore how the offer of a PIS in their language of choice might impact multilingual participants' preferences and their comprehension of the content therein. Further research is required to determine the applicability of these guidelines with more females and also with individuals with more severe NCD symptoms following ABI.

## CONFLICT OF INTEREST STATEMENT

The authors have no conflict of interest to declare.

## Supporting information

Supporting information.

## Footnotes

1 Brookshire, R. H., & McNeil, M. R. (2014). Introduction to neurogenic communication disorders. (8^th^ edition). Elsevier Health Sciences.

2 Brady, M. C., Fredrick, & Williams, B. (2012). People with aphasia: capacity to consent, research participation and intervention inequalities. International Journal of Stroke. 6, 193‐196.

3 Jayes, M. J., & Palmer, R. L. (2014). Stroke research staff's experiences of seeking consent from people with communication difficulties: Results of a national online survey. Topics in Stroke Rehabilitation. 21(5), 443‐451.

4 Shiggins, C., Ryan, B., Dewan, F., Bernhardt, J., O'Halloran, R., Power, E., Lindley, R. I., McGurk, G., & Rose, M. L. (2023). Inclusion of people with aphasia in stroke trials: A systematic search and review. Archives of Physical Medicine and Rehabilitation. 1‐13.

5 Dalemans, R., Wade, D. T., Van den Heuvel, W. J., & De Witte, L. P. (2009). Facilitating the participation of people with aphasia in research: a description of strategies. Clinical Rehabilitation. 23(10), 948‐959; Kagan, A., & Kimelman, M. D. (1995). Informed consent in aphasia research: Myth or reality? Clinical Aphasiology. 23, 65‐75; Pearl, G., & Cruice, M. (2017). Facilitating the involvement of people with aphasia in stroke research by developing communicatively accessible research resources. Topics in Language Disorders. 37(1), 67‐84; Penn, C., Frankel, T., Watermeyer, J., & Müller, M. (2009). Informed consent and aphasia: Evidence of pitfalls in the process. Aphasiology, 23(1), 3‐32; Stein, J., & Brady Wagner, L. C. (2006). Is informed consent a “yes or no” response? enhancing the shared decision‐making process for persons with aphasia. Topics in Stroke Rehabilitation. 13(4), 42‐46.

6 Brennan, A. D., Worrall, L. E., & McKenna, K. T. (2005). The relationship between specific features of aphasia‐friendly written material and comprehension of written material for people with aphasia: An exploratory study. Aphasiology. 19(8), 693‐711; Rose, T. A., Worrall, L. E., Hickson, L. M., & Hoffmann, T. C. (2012). Guiding principles for printed education materials: Design preferences of people with aphasia. International Journal of Speech‐Language Pathology. 14(1), 11‐23.

7 Jayes & Palmer, op. cit. note 3.

8 Johnson‐Greene, D. (2010). Informed consent issues in traumatic brain injury research: current status of capacity assessment and recommendations for safeguards. Journal of Head Trauma Rehabilitation. 25(2), 145‐150; Kompanje, E. J. O., van Dijck, J. T. J. M., Chalos, V., van den Berg, S. A., Janssen, P. M., Nederkoorn, P. J., et al. (2020). Informed consent procedures for emergency interventional research in patients with traumatic brain injury and ischaemic stroke. The Lancet Neurology. 19(12), 1033‐1042.

9 Pearl & Cruice, op. cit. note 5; Penn et al., op. cit. note 5.

10 Dalemans, et al., op. cit. note 5.

11 Jayes & Palmer, op. cit. note 3.

12 Penn, C., & Evans, M. (2009). Recommendations for communication to enhance informed consent and enrolment at multilingual research sites. African Journal of AIDS Research. 8(3), 285‐294.

13 Penn, et al., op. cit. note 5.

14 Frankel, T. (2008). Conversational intelligence after stroke: A drug trial. (Doctoral dissertation). University of the Witwatersrand.

15 Penn, et al., op. cit. note 5.

16 Ibid.

17 Clarke, V., & Braun, V. (2013). Successful qualitative research: A practical guide for beginners. Sage Publishers.

18 Adegboyega, G., Zolo, Y., Arabang Sebopelo, L., Dalle, D. U., Dada, O. E., Bilong Mbangtang, C., Tetinou, F., Kanmounye, U. S., & Folusho Alalade, A. (2021). The burden of traumatic brain injury in Sub‐Saharan Africa: A scoping review. World Neurosurgery. 156, e192‐e205.

19 Matizirofa, L., & Ranganai, E. (2020). An analysis of recent stroke cases in South Africa: Trend, seasonality and predictors. South African Medical Journal. 110(2), 92‐99; Rexrode, K. M., Madsen, T. E., Yu, A. Y., Carcel, C., Lichtman, J. H., & Miller, E. C. (2022). The impact of sex and gender on stroke. Circulation Research. 130(4), 512‐528.

20 Hemispatial neglect = characterised by reduced awareness of stimuli on one side of the body or environment.

21 Fischer, A. E., Venter, W. D. F., Collins, S., Carman, M., & Lalla‐Edward, S. T. (2021). The readability of informed consent forms for research studies conducted in South Africa. South African Medical Journal. 111(2), 180‐183.

22 Pearl & Cruice, op. cit. note 5.

23 Penn, et al., op. cit. note 5.

24 Pearl & Cruice, op. cit. note 5.

25 Yen, P. H., & Leasure, A. R. (2019). Use and effectiveness of the teach‐back method in patient education and health outcomes. Federal Practitioner. 36(6), 284‐289.

26 Sorin‐Peters, R. (2004). The case for qualitative case study methodology in aphasia: An introduction. Aphasiology. 18(10), 937‐949.

27 Shenton, A. K. (2004). Strategies for ensuring trustworthiness in qualitative research projects. Education for Information. 63‐75.

28 Shiggins, C., Ryan, B., O'Halloran, R., Power, E., Bernhardt, J., Lindley, R. I., McGurk, G., Hankey, G. J., & Rose, M. L. (2022). Towards the consistent inclusion of people with aphasia in stroke research irrespective of discipline. Archives of Physical Medicine and Rehabilitation. 103(11), 2256‐2263. (p. 3358)

29 Rose, T. A., Worrall, L. E., Hickson, L. M., & Hoffmann, T. C. (2011). Exploring the use of graphics in written health information for people with aphasia. Aphasiology. 25(12), 1579‐1599.

30 Pearl & Cruice, op. cit. note 5.

31 Rose, et al., op. cit. note 6.

32 Ibid.

33 Shiggins, et al., op. cit. note 4.

34 Jayes & Palmer, op. cit. note 3; Pearl & Cruice, op. cit. note 5.

35 Dalemans, op. cit. note 5; Jayes & Palmer, op. cit. note 3; Kagan, A. (1998). Supported conversation for adults with aphasia: Methods and resources for training conversation partners. Aphasiology. 12(9), 816‐830; Luck, A. M., & Rose, M. L. (2007). Interviewing people with aphasia: Insights into method adjustments from a pilot study. Aphasiology. 21(2), 208‐224; Pearl & Cruice, op. cit. note 5; Penn, et al., op. cit. note 5.

36 Jayes & Palmer, op. cit. note 3.

37 Ibid:165.

