# Supplementary material for: Modifications to consent documentation with adults with communication disorders following brain injury: An exploratory study
Source: Dev World Bioeth. 2024 Jul 14;25(2):118–28. doi: 10.1111/dewb.12458 (PMC12138961; doi:10.1111/dewb.12458)

Supplementary materials

PIS format #1: Extract from a standard Participant Information Sheet using available IRB guidelines from our institution

As you have experienced a stroke or traumatic brain injury in the past, I would like to invite you to volunteer to participate in this research. As part of the research, you will be tested by a neurologist to ensure that the medicine used in this study will not be dangerous for you to take. I will then visit you at your home 4 times to test your thinking skills in different ways. Each of these visits will take about 3 hours of your time. You will have to take videos of yourself having a conversation with a friend or family member that you talk to every day. This will take place 4 times and each recording will be about 30 minutes long. These videos will be used to analyze your conversational skills. Somebody close to you will be asked to fill out a form regarding your communication abilities. They will be asked to fill out the form 4 times. For one month during the study, you will be required to take 2 capsules of medicine called Keppra every day. For another month during the study, you will be required to take 2 capsules a day that contain no medicine. No one will know which month you are taking the capsules with medicine, and which month you are taking the capsules without medicine. This will help to see if Keppra works in improving people's thinking and conversation skills. The study will take 13 weeks in total.

PIS format #2: Extract from a Participant Information Sheet simplified to basic readability level

Because you have had a stroke or traumatic brain injury, I would like to ask you to be a part of this research study. If you choose to be a part of this study, you will have to visit a doctor who will decide if it is safe for you to be a part of the study.

I will visit you at your home 4 times to test your thinking skills. These visits will take 3 hours.

You will take videos of yourself speaking to a friend or family member. You will do this 4 times. I will watch the videos and write about them.

A friend or family member will be asked to fill out a form about how you communicate. They will be asked to fill out the form 4 times.

For one month during the study, you will have to take 2 capsules of medicine called Keppra every day. For another month during the study, you will have to take 2 capsules a day that has no medicine inside. No one will know which month you are taking the capsules with medicine, and which month you are taking the capsules without medicine. This will help to see if Keppra works in helping people communicate better. The study will take 13 weeks.

Keppra might help you to concentrate, remember things and communicate better. It also might not help you.

You may have side effects from taking Keppra. You might feel nervous, have a hard time falling asleep, have sensitive skin, not feel as hungry as usual, feel like you might throw up, feel dizzy, have headaches, feel your heart beating faster, feel more tired than usual, have pain in your stomach, lose weight. Most people do not feel sick when taking Keppra.

PIS format #3: Extract from a Participant Information Sheet simplified to basic readability level with bolding of key information and use of colour

Because you have had a stroke or traumatic brain injury, **I would like to ask you to be a part of this research study**. If you choose to be a part of this study, you will have to visit a **doctor** who will decide if it is safe for you to be a part of the study.

What will you have to do?

1. I will visit you at your home 4 times to **test your thinking skills**. These visits will take 3 hours. You will have to do things like:

- repeat numbers
- write tests
- read
- name colours
- copy pictures
- draw patterns.

2. You will take **videos** of yourself **speaking to a friend or family member**. You will do this 4 times. I will watch the videos and write about them.

3. A friend or family member will be asked to **fill out a form about how you communicate**. They will be asked to fill out the form 4 times.

4. For one month during the study, you will have to take 2 capsules a day of medicine called **Keppra**. For another month during the study, you will have to take 2 capsules a day that have **no medicine inside**. No one will know which month you are taking the capsules with medicine, and which month you are taking the capsules without medicine. This will help to see if Keppra works in helping people communicate better.

The study will take 13 weeks.

PIS format# 4: Extract from a Participant Information Sheet simplified to basic readability level with pictures

What will you have to do?

1. You will see a **doctor** who will decide if it is safe for you to be a part of the research study. The doctor will need your **medical records**

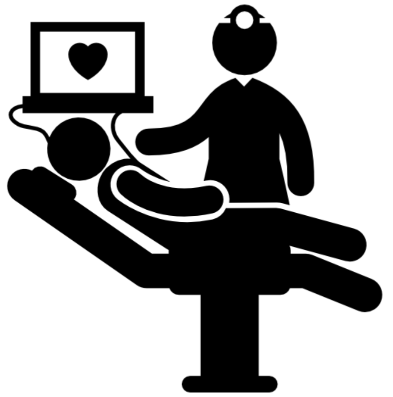

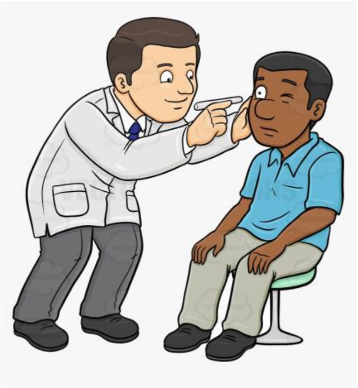

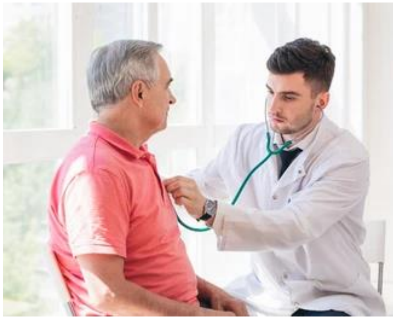

1

2

3

2. I will visit you at your home 4 times to **test your thinking skills**. These visits will take 3 hours.

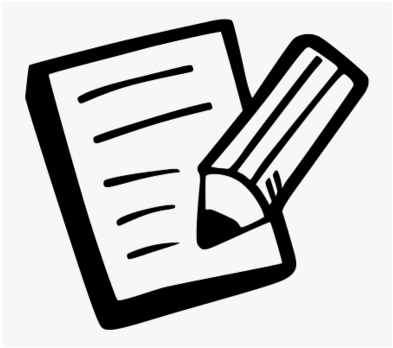

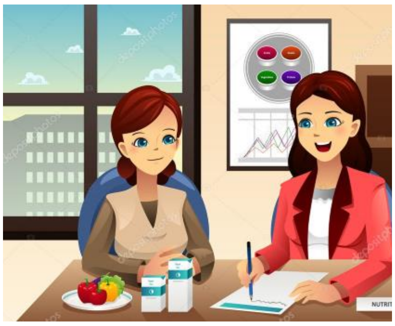

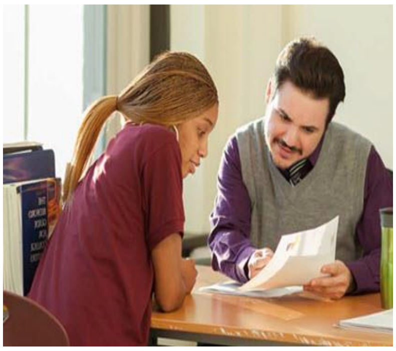

1

2

3

D.

3. You will take **videos** of yourself **speaking to a friend or family member**. You will do this 4 times. I will watch the videos and write about them.

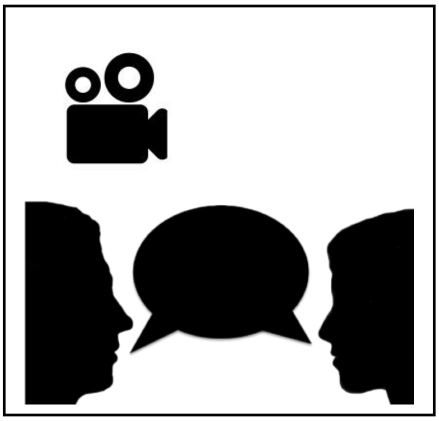

1

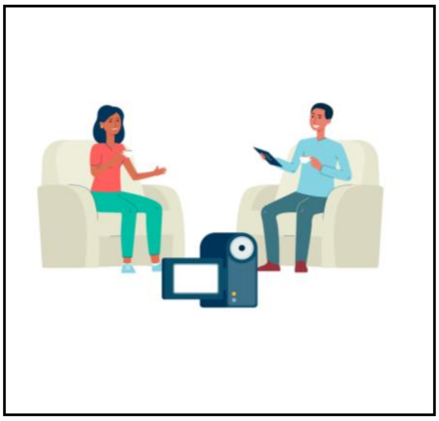

2

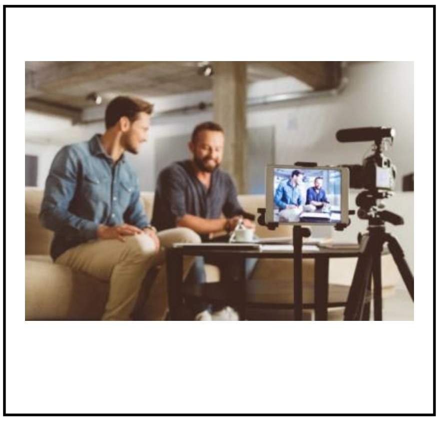

3

PIS format #5: Extract from a PowerPoint presentation giving information about the study using simple language and pictures, presented on an iPad

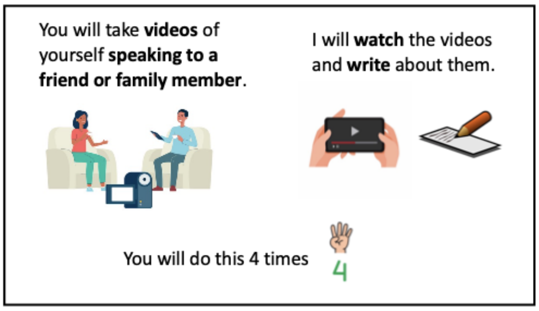

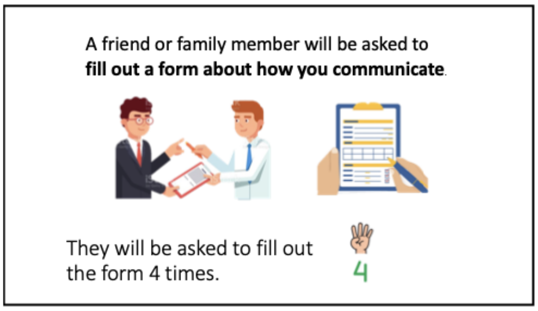

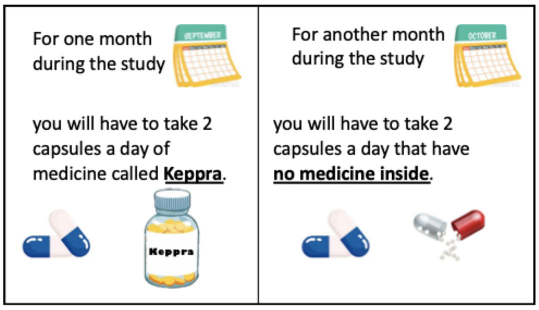

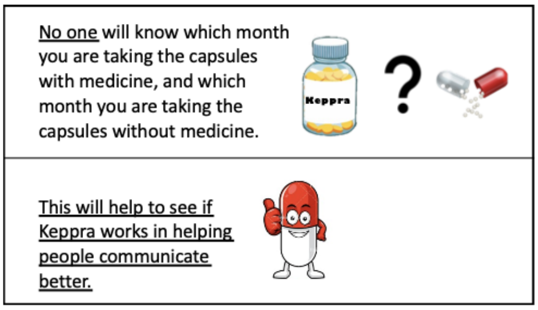

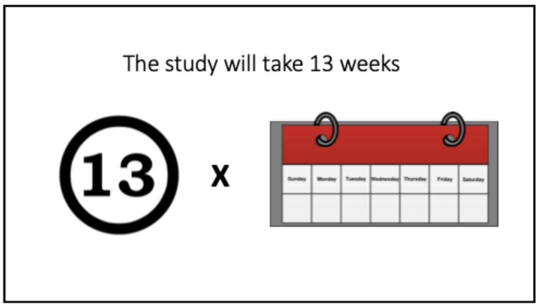

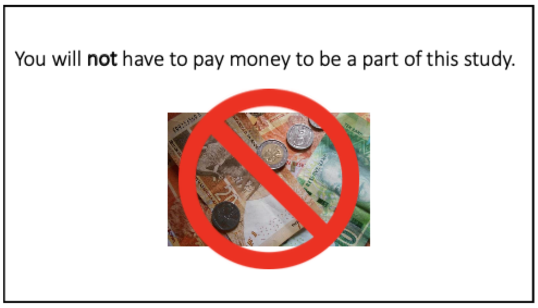

PIS format #6: Extracts from a video recording of the researcher explaining the study, with subtitles and pictures.

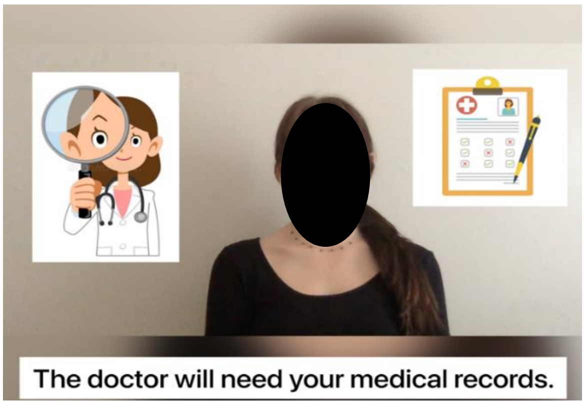

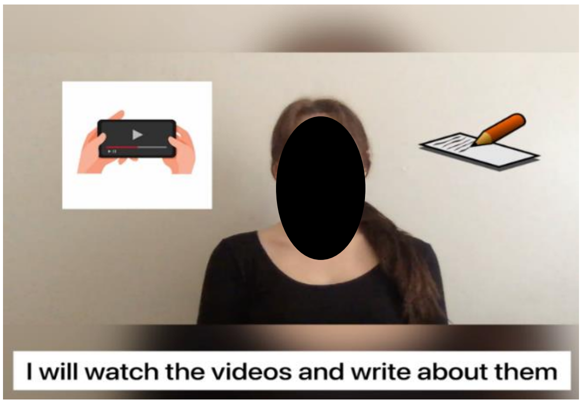

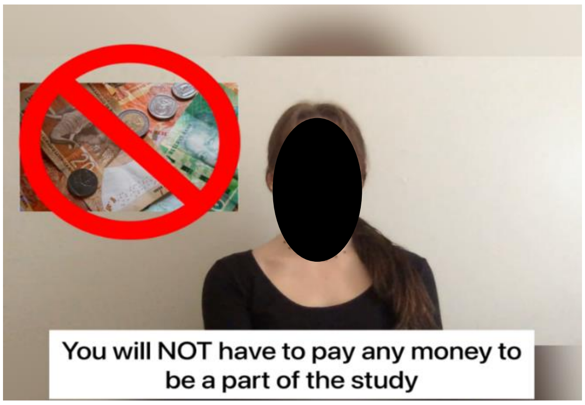

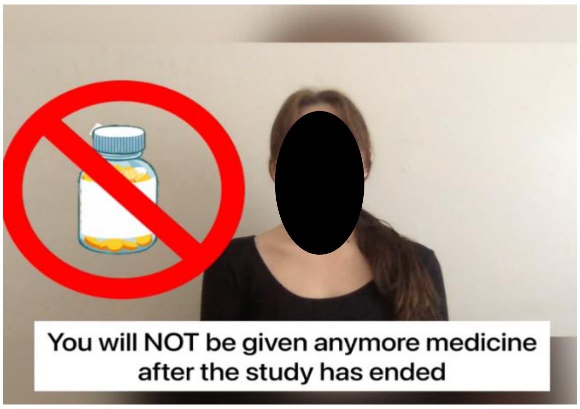

Supplement: Supplementary file 1 — Supporting information. [file DEWB-25-118-s001.pdf]
